# *Fusobacterium* emphysematous pyomyositis with necrotizing fasciitis of the leg presenting as compartment syndrome: a case report

**DOI:** 10.1186/s13256-017-1493-y

**Published:** 2017-11-28

**Authors:** Eduardo Smith-Singares, Joseph Adjei Boachie, Izaskun M. Iglesias, Leland Jaffe, Adam Goldkind, Eric I. Jeng

**Affiliations:** 1 Department of Surgery, Division of Surgical Critical Care, University of Illinois at Chicago, Chicago, 60612 IL USA; 2 Department of Pediatrics, The John H Stroger Hospital of Cook County, Chicago, 60612 IL USA; 3 Department of Surgery, Rosalind Franklin University of Medicine and Science, North Chicago, 60088 IL USA; 4 Department of Surgery, University of Illinois at Chicago, Chicago, 60612 IL USA

**Keywords:** Emphysematous pyomyositis, Necrotizing fasciitis, Compartment syndrome, *Fusobacterium necrophorum*, Case report, Unusual site infection

## Abstract

**Background:**

*Fusobacterium necrophorum* is a common agent of disease in humans, but the occurrence of primary infections outside the head and neck area is extremely rare. While infection with *Fusobacterium necrophorum* has a rather benign course above the thorax, the organism is capable of producing very severe disease when located in unusual sites, including various forms of septic thrombophlebitis. No infections of the leg have been documented before; thus, antibiotic coverage for *Fusobacterium* is currently not recommended in this area.

**Case presentation:**

A 50-year-old homeless African-American man presented complaining of severe pain in his right lower extremity. A clinical workup was consistent with emphysematous pyomyositis and compartment syndrome; he received limb-saving surgical intervention. The offending organism was identified as *Fusobacterium necrophorum*, and the antibiotic coverage was adjusted accordingly.

**Conclusions:**

Bacteria typically involved in necrotizing infections of the lower extremity include Group A ß-hemolytic *Streptococcus*, *Clostridium perfringens*, and common anaerobic bacteria (*Bacteroides*, *Peptococcus*, and *Peptostreptococcus*). This case report presents a case of gas gangrene of the leg caused by *Fusobacterium necrophorum*, the first such case reported. *Fusobacterium* should now be included in the differential diagnosis of necrotizing fasciitis of the extremities.

## Background

Necrotizing infections constitute a variety of different clinical conditions. Early recognition is paramount because the disease can progress to one of massive tissue destruction, systemic toxicity, and even death. Despite early surgical intervention, it has been documented that the rate of amputation and mortality is approximately 50% for both [1]. Patients presenting with necrotizing infections typically have underlying risk factors including diabetes, drug use, obesity, immunocompromised status, recent surgery, or local tissue devitalization [2–4]. Necrotizing fasciitis is often categorized as type I or type II based on the causative bacteria (Table 1) [5]. Bacteria typically involved in necrotizing infections of the lower extremity include Group A ß-hemolytic *Streptococcus*, *Clostridium perfringens*, and common anaerobic bacteria (*Bacteroides*, *Peptococcus*, and *Peptostreptococcus*) [5, 6]. Other anaerobic bacteria commonly produce pathologic processes only above the diaphragm, one of which is *Fusobacterium necrophorum*. There have been no descriptions of *Fusobacterium* infections below the knee in the available English biomedical literature. This case report presents the first confirmed occurrence of necrotizing fasciitis of the lower extremity by *Fusobacterium necrophorum*.

**Table 1:** Epidemiology and risk factors of necrotizing fasciitis

	Type I	Type II
Causative organisms	Aerobic Anaerobic	Group A *Streptococcus* (GAS) Beta-hemolytic streptococci (alone or in combination with other species, most commonly *Staphylococcus aureus*)
Risk factors	Diabetes Peripheral vascular disease (PVD) Immune compromise Recent surgery	Skin injury (laceration or burn) Blunt trauma Recent surgery Childbirth Injection drug use Varicella infection

## Case presentation

**Fig. 1:**
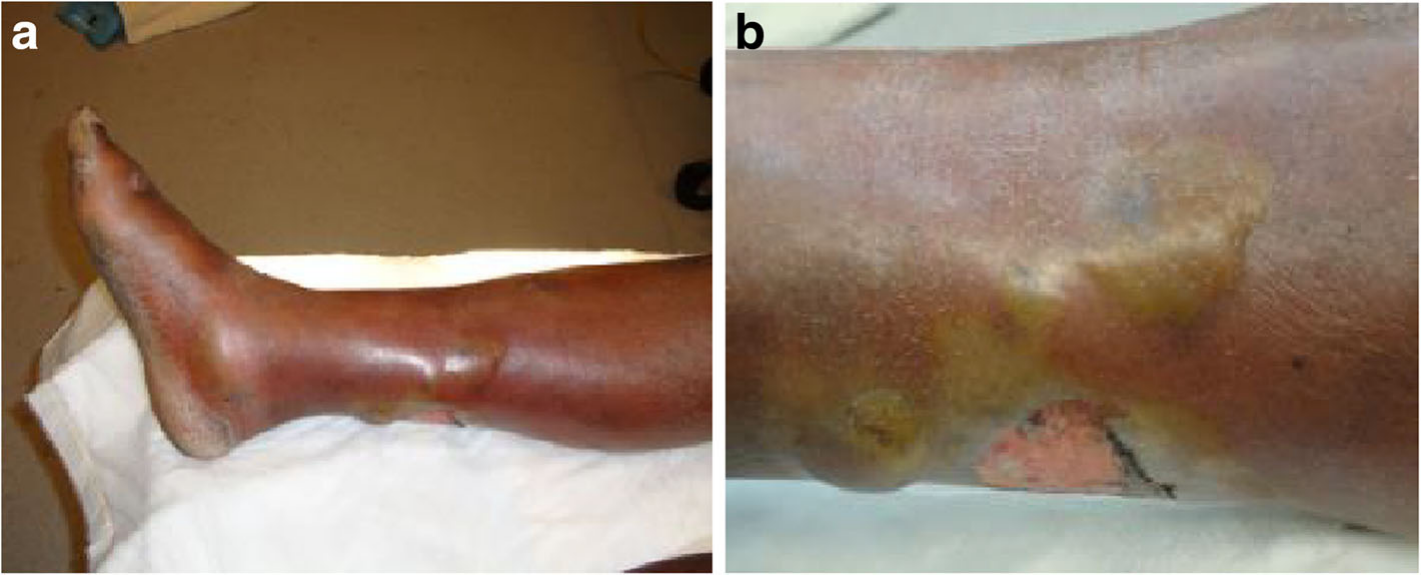
**a** Right lower extremity with multiple serous-filled bullae and a superficial ulceration. **b** Right lower extremity with multiple serous-filled bullae and a superficial ulceration

A 50-year-old African-American man in a state of homelessness presented to the emergency department (ED) at Saint Anthony Hospital in Chicago, a community, faith-based, urban critical access facility staffed by surgeons from the University of Illinois at Chicago (a tertiary, academic center) complaining of severe right lower extremity pain for the past 8 hours. He reported generalized malaise for 3 weeks associated with 2 weeks of productive cough with yellowish sputum and occasional bloody streak, intermittent low grade fevers, nausea, vomiting, and 15 lb (6.8 kg) weight loss. He denied any recent trauma to his leg and has no history of diabetes mellitus. His past medical history was significant only for a previous visit for iron-deficiency anemia. A review of systems disclosed no additional symptoms. He denied any significant family history. He admitted to drinking alcohol in excess, but denied any drinking in the previous 72 hours before presentation. He also admitted to a pack-a-day nicotine habit. He denied current or previous illicit drug use, except for occasional cannabis. A clinical examination showed an African-American man in mild distress. His head and neck examination was only remarkable for partial edentulism and halitosis. His neck had no palpable adenopathies. An examination of his chest showed bilateral crackles at the bases. His heart had a regular rate and rhythm with no murmurs. His abdomen was soft, no masses were palpated. His genitalia appeared normal for his age. His right lower extremity revealed a warm, swollen, erythematous leg with multiple serous-filled bullae on the dorsolateral and posterior aspect, and a superficial ulceration at the posterior aspect (Fig. 1a and b). His neurological examination showed a patient awake, alert, and oriented times four (person, place, time, and situation). A cranial nerve examination was normal. No sensory or motor deficits (other than those described above) were detected. The rest of his physical examination was unremarkable.

### Diagnostic assessment

**Fig. 2:**
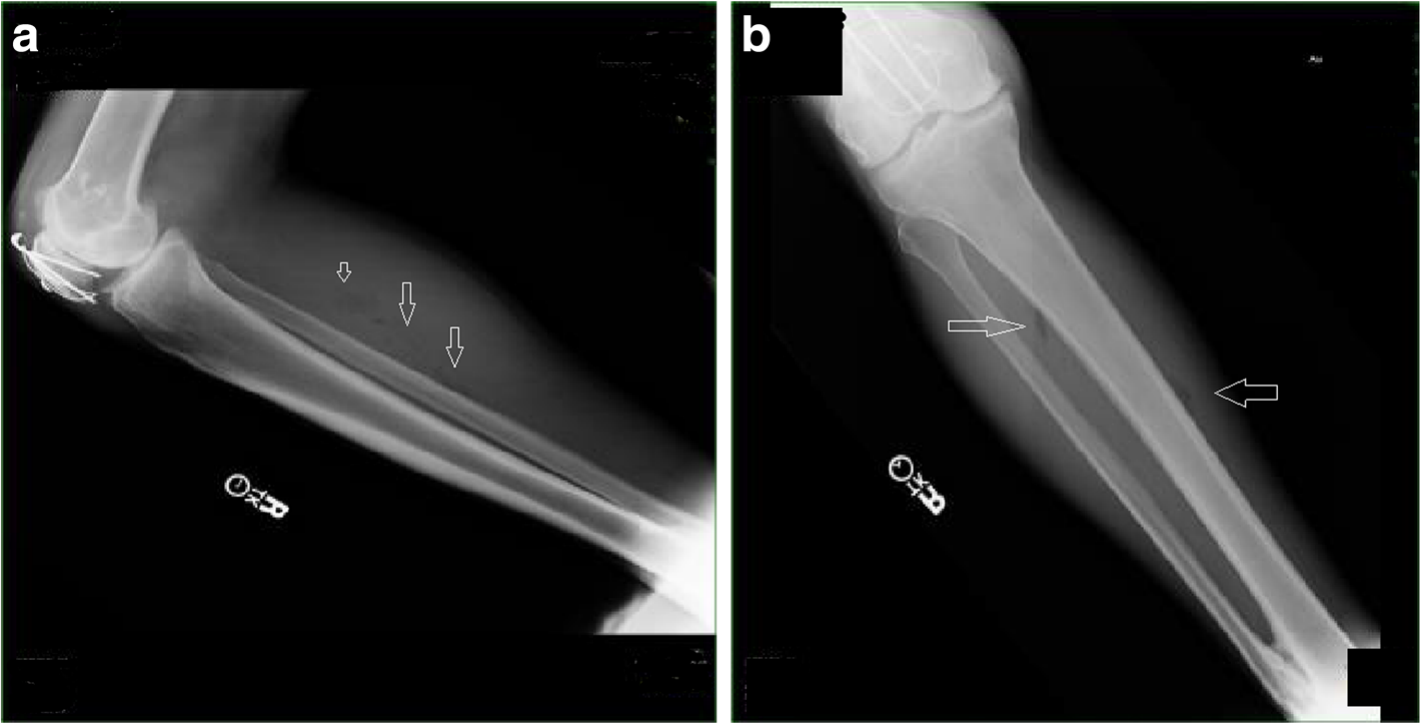
**a** Subcutaneous emphysema of the deep posterior compartment of the right lower extremity. **b** Subcutaneous emphysema of the deep posterior compartment of the right lower extremity

Initial laboratory values of white blood cell (WBC) count of 24.0 per mm^3^, hemoglobin of 13.4 g/dL, serum sodium level of 131 mEq/L, serum creatinine of 2.89 mg/dL, C-reactive protein 199 mg/L, and serum glucose of 120 mg/dL were strongly suggestive of necrotizing fasciitis based on the Laboratory Risk Indicator for Necrotizing Fasciitis (LRINEC) score (Table 2) [7]. His LRINEC score was found to be 10. A chest X-ray was consistent with pneumonia, while the X-rays of his right lower extremity revealed subcutaneous emphysema of the deep posterior compartment of the right leg (Fig. 2a and b). Purified protein derivative (PPD) was negative.

**Table 2:** Laboratory Risk Indicator for Necrotizing Fasciitis score

Variable, units	Score
C-reactive protein, mg/L	
< 150	0
≥ 150	4
Total white cell count, per mm^3^	
< 15	0
15–25	1
> 25	2
Hemoglobin, g/dL	
> 13.5	0
11–13.5	1
< 11	2
Sodium, mmol/L	
≥ 135	0
< 135	2
Creatinine, mg/dL	
≤ 1.6	0
> 1.6	2
Glucose, mg/dL	
≤ 180	0
> 180	1

The maximum score is 13; a score ≥ 6 raises suspicion of necrotizing fasciitis and a score ≥ 8 is strongly predictive of the disease

### Therapeutic interventions

**Fig. 3:**
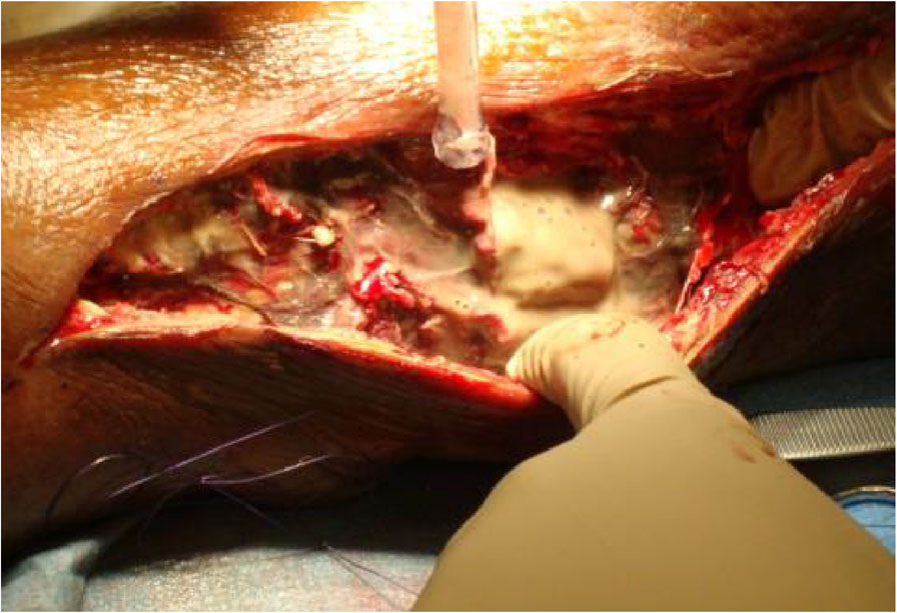
Purulent drainage was expressed from the deep posterior compartment of the right lower extremity

He was started on broad spectrum antibiotics (vancomycin and piperacillin/tazobactam) and was taken to the operating room, where he underwent a four-compartment fasciotomy of his right lower extremity with extensive debridement of necrotic fascia and muscle. A medial incision was made from a point distal to the tibial plateau and extended to his ankle joint. Extensive purulent drainage was immediately noticed in the subcutaneous tissues. The medial fascia of the posterior and deep posterior compartments was sectioned and approximately 500 mL of purulent drainage was suctioned from the deep posterior compartment of his leg (Fig. 3). The gastrocnemius and soleus muscle bellies were edematous but viable. A lateral incision was then made from a point midway between the tibial plateau and the fibular head, and extended distally to his ankle joint. The lateral incision was dissected through the subcutaneous tissues until the fascia of the anterior and lateral compartments was visualized. The muscle bellies of the anterior and lateral muscle groups were also edematous but viable with no evidence of necrosis or purulent drainage from these compartments. A fasciotomy was also performed at the plantar and dorsolateral aspect of his right foot.

### Microbiology workup

All surgical samples were collected using an eSwab Transport System (CoPan Diagnostics Inc., Murrieta, CA, USA), which handles all aerobic, anaerobic, and fastidious microorganisms. Blood cultures were placed in designated aerobic and anaerobic blood culture bottles (BacT/ALERT, Durham, NC, USA). The clinical laboratory at Saint Anthony Hospital outsources its microbiology to another laboratory in the Chicago area (LabCorp, 321 W Lake Street Suite C, Elmhurst IL, 60126). After samples are received from the operating room, they are packaged as per LabCorp specifications, and a courier is summoned. The estimated travel time is less than 4 hours. For surgical samples two different media cultures are used: for aerobic samples the swab containing the specimen is immersed in a gel-based transfer media; for anaerobic samples the Port-A-Cul transport system (BD – Beckton Dickinson, Franklin Lakes, NJ, USA) is used. Once the samples arrive at LabCorp they are processed as per proprietary procedures. Anaerobes in this sample were identified using RapID-ANA Kit (Thermo Fisher Scientific, Waltham, MA, USA). In addition, LabCorp results confirmed the presence of beta-hemolysis in the cultured plates.

Initial Gram stains of the pus collected showed only rare WBCs and no microorganisms. Blood cultures taken prior to surgical intervention (both bottles) and cultures from his right lower extremity wounds following the initial fasciotomy demonstrated *Fusobacterium necrophorum*, which was beta-lactamase negative and sensitive to cefoxitin, chloramphenicol, clindamycin, penicillin, doripenem, and metronidazole (determined by broth microdilution). No additional microorganisms were identified. The sputum cultures showed no growth.

### Follow ups and outcome

**Fig. 4:**
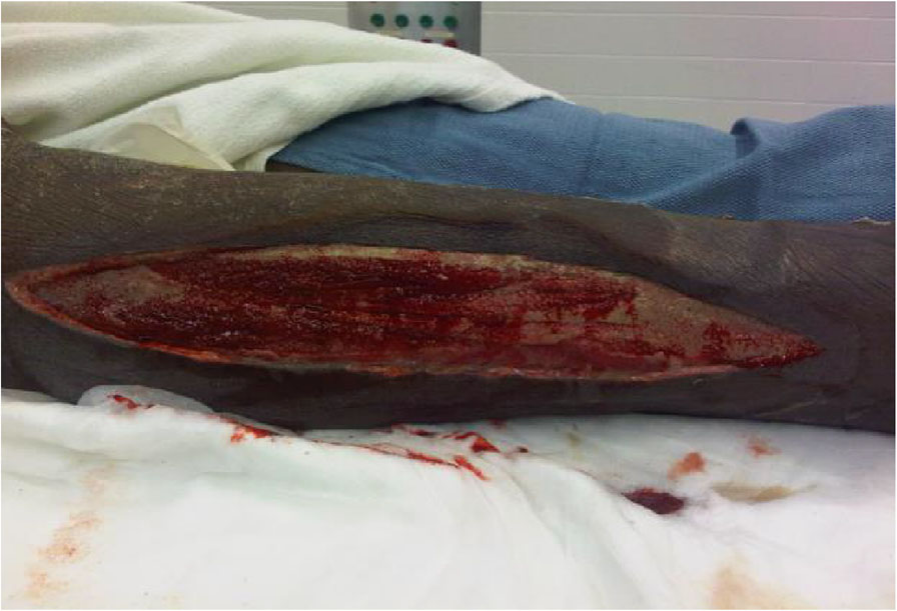
Granulation tissue covering the base of the wound after multiple debridements

He was transferred postoperation to the general surgical floor, where the remainder of his hospital course consisted of multiple serial surgical debridements, pulse lavage of his wounds, and application of wound V.A.C.® dressing changes every 2 to 3 days. The wound care team at Saint Anthony Hospital (consisting of podiatric surgeons and residents from the Rosalind Franklin University of Medicine and Science) was consulted to assist with the management of the fasciotomy wounds. He improved clinically and the wound granulated over the next 8 weeks (Fig. 4). He subsequently underwent split-thickness skin grafting and was discharged for continued out-patient management to a local shelter facility. After a few late visits and some missing appointments, he was lost to follow up.

## Discussion

This case report presents a rare type I idiopathic necrotizing lower extremity infection from *Fusobacterium necrophorum*: Gram-negative, obligate anaerobe, non-spore forming, pleomorphic bacillus [8]. It has been isolated from the normal flora in the oral cavity, gastrointestinal tract, and genitourinary tract [9]. When involved in disease and infection, necrotic lesions and deep abscess formation can occur, and bacteremia is not uncommon [10]. *Fusobacterium necrophorum* is a well-established agent of disease above the diaphragm: it is commonly associated with Lemierre’s syndrome, a septic infection caused by thrombose formation within the jugular vein after colonization of a peritonsillar abscess [11–13]. In recent epidemiological surveillance studies, *Fusobacterium* has been determined to be the predominant organism causative of pharyngitis in a university clinic with 21% of the cases [11]. Much more rarely though, *Fusobacterium necrophorum* has been described as a potential causative agent of infections below the diaphragm. Beldman *et al*. described a case of septic arthritis of the hip caused by *Fusobacterium necrophorum* following a tonsillectomy [14] and Patel *et al*. also reported in an abstract a case of necrotizing fasciitis and pyomyositis in the thigh caused by *Fusobacterium necrophorum* in a healthy adult [15]. To the best of our knowledge, no other reports have been described directly linking *Fusobacterium necrophorum* as the causative organism for necrotizing infections below the knee in the literature. Early diagnosis and treatment is critical due to the rapid extensive tissue destruction that ensues with these infections, and thus maintaining a high index of suspicion is vital for the survival of these patients. A high index of suspicion is required when choosing antibiotic coverage for necrotizing fasciitis and pyomyositis, and additional case reports of this occurrence may define a pattern of risk factors that should prompt *Fusobacterium* coverage.

Our patient did not have any of the described risk factors for necrotizing fasciitis: diabetes mellitus, documented instances of recent and chronic intravenous drug abuse, age greater than 50, hypertension, and malnutrition/obesity [16]. There were no obvious entrance wounds; thus, hematogenous spread of the infection from a possible pneumonic source or from oropharyngeal foci could not be ruled out, and has been described before [17, 18].

In the preparation of this case report the CARE guidelines were followed (surgical extension, which is available at http://www.scareguideline.com; the SCARE checklist is available in Table 3 of [19]).

## Conclusions

*Fusobacterium necrophorum* which is often associated with Lemierre’s syndrome and fasciitis, pyomyositis, or osteomyelitis occurring above the diaphragm, can also cause necrotizing infections of the lower extremities. Treatment involves early and aggressive surgical exploration, debridement of necrotic tissue, hemodynamic support, and antibiotics.

## Patient’s perspective

Since the patient was lost to follow up, an updated perspective of his previous and current condition is impossible.
